# Apolipoprotein E4 in Alzheimer’s Disease: Role in Pathology, Lipid Metabolism, and Drug Treatment

**DOI:** 10.3390/ijms27021004

**Published:** 2026-01-19

**Authors:** Nour F. Al-Ghraiybah, Amer E. Alkhalifa, Yutaka Itokazu, Taylor O. Farr, Naima C. Perez, Hande Ali, Amal Kaddoumi

**Affiliations:** 1 Department of Pharmacology and Toxicology, Medical College of Georgia, Augusta University, Augusta, GA 30912, USA or nfa0007@auburn.edu (N.F.A.-G.); or aea0068@auburn.edu (A.E.A.); yitokazu@augusta.edu (Y.I.); tfarr@augusta.edu (T.O.F.); naima.c.perez@gmail.com (N.C.P.); 2 Department of Drug Discovery and Development, Harrison College of Pharmacy, Auburn University, Auburn, AL 36849, USA; 3 Faculty of Pharmacy, Zonguldak Bülent Ecevit University, Zonguldak 67100, Türkiye; hande.dos@ef.karaelmas.edu.tr

**Keywords:** Alzheimer’s disease, Apolipoprotein E, ApoE4, amyloid-β, blood–brain barrier, lipid metabolism, cholesterol, monoclonal antibodies, precision medicine

## Abstract

Alzheimer’s Disease (AD) is a neurodegenerative disorder characterized by cognitive decline and memory loss. Among the genetic risk factors linked to AD, the Apolipoprotein E4 (ApoE4) remains the strongest. It is well known that carrying the ApoE4 isoform is associated with advanced AD pathology, blood–brain barrier (BBB) disruption, and changes in lipid metabolism. In this review, we provide an overview of the role of centrally and peripherally produced ApoE in AD. After this introduction, we focus on new findings regarding ApoE4’s effects on AD pathology and BBB function. We then discuss ApoE’s role in lipid metabolism in AD, highlighting examples of lipid changes caused by carrying the ApoE4 isoform. Next, the review explores the implications of ApoE4 isoforms for current treatments—whether they involve anti-amyloid therapy or other pharmacological agents used for AD—emphasizing the importance of personalized medicine approaches for patients with this high-risk allele. This review aims to provide an updated overview of ApoE4’s effects on AD pathology and treatment. By integrating recent discoveries, it underscores the critical need to consider ApoE4 status in both research and clinical settings to enhance therapeutic strategies and outcomes for individuals with AD.

## 1. Introduction

Alzheimer’s Disease (AD) is a neurodegenerative disorder characterized by cognitive decline and memory loss, accounting for 60–80% of dementia cases in the United States. It is estimated that in 2025, approximately 7.2 million Americans aged 65 and older will live with the disease. The pathological features of AD include brain atrophy, neuroinflammation, extracellular amyloid-β (Aβ) accumulation, and intracellular hyperphosphorylated tau, which manifests as neurofibrillary tangles (NFT) [1,2]. AD is classified into two types: early-onset Alzheimer’s Disease (EOAD) and late-onset Alzheimer’s Disease (LOAD). EOAD affects people under 65 years old, makes up 2–10% of cases, and is associated with autosomal dominant mutations in the amyloid precursor protein (APP), presenilin 1 (PSEN1), and presenilin 2 (PSEN2) genes [2,3]. In contrast, LOAD affects individuals aged 65 and older. It is partly linked to the Apolipoprotein E (ApoE) gene and its isoforms, which remain the leading genetic risk factor for developing LOAD [4]. AD has several risk factors, including modifiable factors like diet and sleep patterns, as well as non-modifiable factors such as sex and ApoE isoforms.

### 1.1. Modifiable Risk Factors

Diet significantly influences the pathogenesis of AD, although the precise mechanisms remain elusive. Notably, nutritional patterns such as the Mediterranean Diet-low in red meats, moderate in fish, poultry, and alcohol, and rich in whole grains, fruits, vegetables, nuts, legumes, and olive oil-exhibit anti-inflammatory effects and are associated with enhanced cognitive function, a reduced rate of cognitive decline, and a lower incidence of cognitive impairment [5]. Supporting the role of diet, our lab recently found that daily consumption of extra-virgin olive oil in patients with mild cognitive impairment improved behavioral scores, enhanced brain connectivity, and reduced blood–brain barrier (BBB) permeability [6]. Furthermore, in a comprehensive review, Zhang et al. showed that diets abundant in omega-3 fatty acids correlate with a diminished risk of developing AD [7]. Conversely, diets rich in saturated fats and trans fats, coupled with low levels of antioxidants, have been associated with an elevated risk of AD [8].

Among other modifiable risk factors for AD currently being studied, inadequate sleep has attracted significant attention. Research shows that individuals with AD have more sleep disturbances than age-matched controls [9]. In humans, sleep deprivation has been shown to modify plasma levels of inflammatory markers and increase CSF biomarkers indicative of neuronal injury [10,11]. Given that one of sleep’s critical roles is the clearance of neurotoxic Aβ and other metabolic byproducts from the central nervous system (CNS), impaired sleep quality could exacerbate Aβ accumulation and thereby elevate the risk of both the onset and progression of AD [10].

### 1.2. Non-Modifiable Risk Factors

Approximately two-thirds of individuals diagnosed with AD in the United States are females, indicating a strong correlation between sex and AD pathology [7]. This disparity may be attributed to sex-specific risk factors, such as the subsequent decline in estrogen levels and the physiological influences of menopause [12,13]. The menopause transition (MT) in women is characterized by decreased ovarian function and impaired estrogen-regulated systems, such as thermoregulation [14]. Among the hormones dysregulated during MT is estradiol, which has confounding effects on inflammation and oxidative stress [14]. Neurologically, healthy women exhibit lower synapse density, higher tau, and higher Aβ compared to their age-matched male counterparts, and exhibit faster atrophy once neurodegeneration begins [15].

ApoE is a 34 kDa glycoprotein that is involved in many biological processes, including plasma lipoprotein metabolism, intracellular cholesterol utilization, cell growth, immunoregulation, and neuronal growth and repair [16,17]. ApoE is produced both in the periphery and in the brain. The peripheral and CNS ApoE pools are mostly kept separated by the BBB [17]. Within the CNS, ApoE is predominantly produced by astrocytes, with contributions also made by other glial cells, especially microglia [18].

ApoE is the main apolipoprotein present in the CNS and is involved in brain vasculature regulation in both the presence and absence of neurodegeneration-related pathology [18,19]. The three common isoforms of human ApoE are ApoE2, ApoE3, and ApoE4 [20], with ApoE4 being the strongest genetic risk factor for AD, increasing the risk by 3–4 times (with one copy) to 9–15 times (with two copies) [1,3]. ApoE4 is implicated in 60–80% of AD cases and lowers the age of onset [1,3]. ApoE2 differs from ApoE3 by the single amino acid substitution at Arg158Cys, whereas ApoE4 differs from ApoE3 by the single amino acid substitution at Cys112Arg. The amino acid substitution enhances ApoE4’s lipid-binding ability [21]. ApoE2 confers a protective effect and is associated with the lowest risk of AD development (~14%) compared to ApoE4 (~28%) [1,18,20,22]. Moreover, Neu et al. reported that, among individuals aged 55–85 with the ApoE3/E4 genotype, the odds of developing AD were roughly equivalent between both sexes. However, female ApoE4 carriers aged 65 to 75 exhibited an increased risk for AD compared to their male counterparts [12,23]. Additionally, research suggests that males with a single copy of the ApoE4 allele do not demonstrate an elevated risk compared to non-carriers [7].

Furthermore, interactions between ApoE isoforms and modifiable risk factors are described as a gene–environment interplay that can influence AD pathology [24]. ApoE isoforms may interact with dietary factors, thereby affecting glucose metabolism, body weight, and neurodegeneration [25]. For example, positive effects of omega-6 intake have been observed in ApoE4 carriers compared to non-carriers [26]. Additionally, it has been reported that ApoE4 could impact the sleep quality [27]. For example, ApoE4 carriers tend to experience shorter rapid eye movement (REM) sleep, more sleep disturbances, and increased fragmentation of slow-wave sleep compared to non-ApoE4 carriers [28,29].

In this review, we examine new findings regarding the role of the ApoE4 isoform in AD pathology, with a particular focus on BBB disruption and lipid metabolism. Subsequently, we provide an overview of the ApoE4 isoform’s influence on treatment efficacy in AD.

## 2. Methods

This narrative review offers a thorough and qualitative overview of the current literature. Our method involved searching scholarly databases, including recent research on ApoE isoforms and clinical trial results. Specifically, we searched PubMed and Google Scholar, limiting our search to English-language publications. The keywords used included Alzheimer’s disease, Alzheimer’s risk factors, Apolipoprotein E, Blood-brain Barrier, Lipidomics, aducanumab, lecanemab, donanemab, and amyloid-related imaging abnormalities. Titles and abstracts were carefully screened for relevance.

## 3. ApoE4 Isoform in AD Pathology

ApoE4 exerts several pathogenic effects on AD progression. These effects may arise from peripheral ApoE, which contributes to systemic metabolic and inflammatory disturbances relevant to AD progression, or from CNS-produced ApoE. In this section, we provide an overview of ApoE4’s deleterious effects on AD pathology.

### 3.1. Peripheral ApoE Role in AD Pathology

Although peripherally and centrally produced ApoE are two separate pools, the peripheral ApoE isoform can exert central effects on AD pathology [17,30,31]. In general, it has been suggested that lower plasma levels of ApoE are associated with cognitive decline in WT mice, as ApoE might play a role in neuronal signaling by preserving the synapto-dendritic complex [32]. Similarly, human data showed that lower plasma ApoE levels, regardless of specific isoform, correlated with cognitive decline in MCI [33]. In the study cohort, the ApoE4 genotype was associated with lower plasma ApoE levels, whereas ApoE2/3 was associated with the highest plasma ApoE levels [33]. Notably, plasma ApoE levels declined with age in ApoE4 carriers, with a significantly greater reduction in females than in males, which might, in part, explain the higher AD risk in females [33]. Low plasma ApoE levels are associated with brain structural changes, as lower levels are associated with reduced hippocampal volume in cognitively normal subjects and MCI patients [30]. ApoE levels and hippocampus volume association were observed irrespective of age, sex, and ApoE genotype, suggesting that peripheral ApoE may exert its effect regardless of ApoE4 isoform [30]. In the same study, the authors did not find a link between peripheral ApoE levels and hippocampal volume in the AD group; the absence of association was attributed to “early dynamic decline,” which reaches the “floor” in the early MCI stage of AD [30]. Liu et al. have evaluated the specific role of peripheral ApoE4 on brain function by using a conditional knock-in mouse model of human ApoE in the liver [31]. In this study, peripheral ApoE4 exacerbates AD pathology and impairs cognition by compromising cerebrovascular function compared with the ApoE3 genotype [31]. Peripheral ApoE4 increased the BBB permeability by altering tight junction proteins, reduced cerebral blood flow rate in the cortical arterioles, and increased gliosis [31]. Single-cell transcriptomic analysis of astrocytes revealed that peripheral ApoE4 can induce changes, such as downregulation of astrocyte end-feet markers and increased immune responses [31]. Another study suggests that peripheral ApoE can influence CNS ApoE by crossing the BBB through serum exosomes [34]. This study, conducted in humans, demonstrated that aged or AD subjects had higher ApoE4 levels in serum exosomes compared to young, non-AD subjects [34]. Those ApoE4-laden exosomes can cross the BBB, and their presence is associated with an age-dependent decrease in thyroid hormone and cognitive decline [34]. The study authors explained that ApoE4-carrying exosomes can cross the BBB transcellularly through the choroid plexus and distribute in the brain, causing increased intracellular neuronal cholesterol levels, which ultimately lead to heightened oxidative stress and activation of the nucleotide-binding oligomerization domain-like receptor family pyrin domain-containing 3 (NLRP3) inflammasome, resulting in neuroinflammation and neuronal dysfunction [34].

### 3.2. CNS ApoE Role in AD Pathology

ApoE isoform exerts broad effects on both Aβ and tau pathology. In the human brain, carrying the ApoE4 genotype is associated with higher brain Aβ burden, as measured by ^18^F-florbetaben PET scan in diffuse cortical regions, and with higher tau burden, as measured by ^18^-flortaucipir PET scan in the lateral and medial temporal, cingulate, and insula cortices [35]. Following up with the patients for two years, ApoE4 carriers had faster tau accumulation across multiple regions, which remained significant after controlling for baseline Aβ load, whereas, although Aβ load modestly increased with time, it generally lost significance after multiple-comparison correction [35]. These results suggest that ApoE influences both pathologies; however, its impact on Aβ appears stage-dependent and weaker than its robust effect on tau pathology [35].

When evaluating the role of ApoE4 in Aβ and tau pathology, multiple mechanisms have recently been proposed. Neuronal ApoE4 can activate transcription factor CCAAT/enhancer-binding protein β (C/EBPβ); the activation of this transcription factor can upregulate APP, tau, and β-site APP-cleaving enzyme 1 (BACE1) mRNA expression in a maturation-dependent manner in SH-SY5Y neuronal cells and mouse primary neurons [36]. These findings were replicated in mature neurons differentiated from human induced pluripotent stem cells (iPSCs) derived from patients with AD, but not in neurons from healthy controls [36]. Blocking the ApoE receptor with RAP (receptor-associated protein) inhibited this signaling pathway and reduced ApoE4-induced proinflammatory cytokines in primary neuronal cells. Later, the authors developed a neuronal-specific Thy1-ApoE4/C/EBPβ double transgenic mouse model to evaluate the role of neuronal ApoE4 in AD pathology; these mice exhibited abnormal phenotypes, including reduced body weight, agitation, and cognitive deficits [36]. Moreover, these mice exhibited Aβ deposits, NFTs, heightened neuroinflammation, and neurodegeneration, which collectively suggest that neuronal ApoE4 genotypes can directly induce both AD pathologies: tau and Aβ [36]. Furthermore, the ApoE4 genotype is associated with impaired cognitive performance, as assessed by the Mini-Mental State Examination (MMSE). This association is mediated through the activation of glycogen synthase kinase-3β (GSK-3β) in patients with type II diabetes, indicating the potential role of ApoE4 in AD independent of amyloidopathy or tauopathy [37]. When assessing the role of the human ApoE genotype in 5xFAD AD mice, carrying the ApoE4 genotype accelerated amyloid deposition and microglia activation starting from the presymptomatic phase at three months old, compared to 5xFAD mice with the ApoE3 isoform [38]. Moreover, proteomic and biochemical analyses revealed an ApoE4-associated mitochondrial dysfunction where HT22 mouse hippocampal neuronal cells exposed to ApoE4 showed increased mitochondrial fusion and mitophagy, and reduced mitochondrial fission, compared to ApoE3-treated cells, suggesting ApoE4’s role in altering energy metabolism, and highlighting the deleterious effects of ApoE4 at the presymptomatic stage of AD [38]. ApoE4-induced alterations in mitochondrial dynamics are expected to increase reliance on lysosome-mediated mitophagy for the removal of dysfunctional mitochondria, thereby functionally linking mitochondrial defects to lysosomal clearance capacity. It has been shown that astrocytic ApoE4 can increase glycolytic activity, impair mitochondrial respiration, and induce cholesterol accumulation. These ApoE4-related changes impair the removal of damaged mitochondria through lysosomes, suggesting the direct link between mitochondrial dynamics and lysosomal alterations in ApoE4 astrocytes [39]. Another recent study has revealed that ApoE4 can alter lysosomal activity in neurons [40]. Lysosomal function in transfected neuronal cells (murine neuroblastoma cell line Neuro-2a) that were engineered to express human ApoE3 or ApoE4 was affected. ApoE4 cells exhibited decreased lysosomal proteolytic activity, indicating a reduced capacity to degrade proteins and damaged organelles compared to ApoE3 cells [40]. Moreover, ApoE4 cells had less efficient mitophagy. Collectively, these findings suggest that neuronal damage in individuals with the ApoE4 genotype might arise from defective lysosomal degradation, and that restoring lysosomal acidity and functionality might help preserve neuronal function [40]. Similarly, another study showed that ApoE4 disrupts autophagic and lysosomal processing in vitro by causing ApoE4 accumulation in enlarged lysosomes, thereby impairing autophagic flux [41]. These defects suggest that ApoE4 compromises intracellular clearance pathways, adding another layer to its contribution to neuronal vulnerability [41].

ApoE can directly interact with Aβ proteins [42]. Single-molecule imaging investigations have demonstrated that all ApoE isoforms, encompassing both lipidated and non-lipidated forms of ApoEs, directly interact with Aβ proteins during the initial stages of Aβ aggregation [42]. ApoE-Aβ aggregates are composed of 20–50% ApoE. ApoE isoforms did not alter the kinetics of Aβ aggregation; however, non-lipidated ApoE4-Aβ aggregates were the slowest to be cleared via glial cells [42]. These results suggest that non-lipidated ApoE4 may, at least in part, drive early Aβ aggregation, offering a new target for early treatment of Aβ pathology in ApoE4 carriers [42]. Similarly, recent cellular data support an intracellular interaction between ApoE and Aβ [43]. Astrocytic ApoE can get internalized by neurons and colocalizes with Aβ and APP within neuronal endosomes and autophagosome-like compartments of neurites [43]. Moreover, neurons exposed to ApoE4 show elevated levels of endogenous and internalized Aβ_42_ compared with those exposed to ApoE3, suggesting that ApoE4 may influence extracellular and intracellular Aβ aggregation [43]. ApoE can also directly interact with tau. Specifically, the ApoE3 R136S, or Christchurch, variant was found to bind strongly to tau and modulate its cellular uptake, cleavage, and propagation [44]. ApoE3 R136S variant is a PSEN1 E280A carrier for the ApoE3 Christchurch variant, which is protective against AD [44]. Published work has shown that in the ApoE3 R136S variant, the ApoE binds to tau to reduce neuronal and microglia uptake compared to wild-type ApoE3, reducing tau fragmentation and neurotoxicity in vitro in primary cortical neurons and in vivo in 5xFAD and Tau P301S mice [44].

### 3.3. Role of ApoE Isoforms on the BBB Integrity

ApoE isoform is strongly associated with BBB dysfunction, an early pathological event in AD [45,46]. The BBB is a semi-permeable neurovascular interface that regulates the transport of substances between the cerebral blood and the parenchymal extravascular space. The BBB comprises endothelial cells supported by pericytes and astrocytes. Collectively, these components uphold brain homeostasis by preventing the infiltration of blood-borne toxins, selectively mediating the transport of nutrients, and facilitating waste removal [47,48,49]. The endothelial cells constitute the physical barrier, with these cells interconnected through tight junction proteins, including claudin-5 and occludin, as well as by cytoplasmic scaffold proteins such as zona occludens (ZO) proteins. These components are crucial for limiting paracellular transport [50]. Furthermore, astrocytes contribute through their end-feet, which encircle blood vessels and help regulate ion and water balance. They express the aquaporin-4 (AQP4) channel and secrete factors that are instrumental in maintaining the integrity of the BBB [51]. Pericytes contribute to vascular stability, regulate capillary blood flow, control BBB permeability, and modulate the expression of BBB-specific proteins in endothelial cells [52,53]. BBB key functions encompass the regulation of nutrient entry, such as glucose through glucose transporter 1 (GLUT1), the removal of metabolic waste, including Aβ via the low-density lipoprotein receptor-related protein 1 (LRP1), and the efflux transporter P-glycoprotein (P-gp). Additionally, it involves the prevention of pathogen and neurotoxic substance entry and the preservation of ionic and osmotic balance, which are vital for neuronal signaling [45]. In AD, the BBB integrity and function are compromised. Primarily, the expression of tight junctions in endothelial cells is diminished, and pericytes degenerate. Furthermore, transport systems responsible for Aβ clearance are compromised, as evidenced by decreased levels of LRP1 and P-gp, and by an increased influx mediated by the Receptor for Advanced Glycation End Products (RAGE) [54]. The defective BBB contributes to the accumulation of Aβ in the brain parenchyma, neuroinflammation, oxidative stress, and ultimately, neuronal injury and cognitive decline in AD [55].

**Figure 1:**
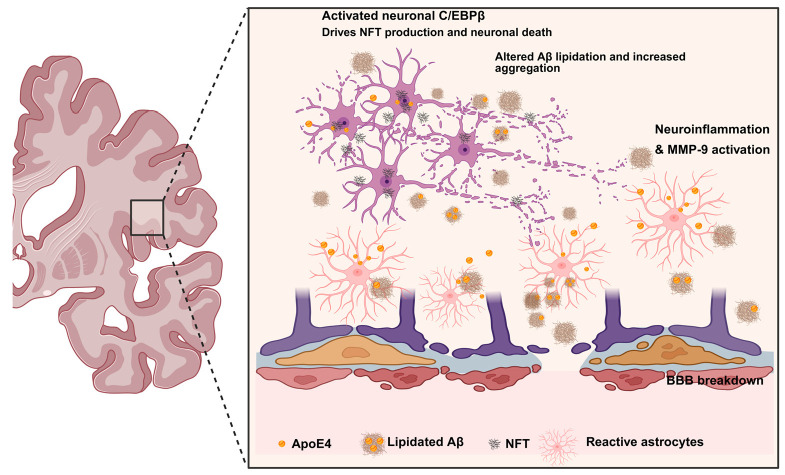
ApoE4 isoform plays a significant role in AD pathology. ApoE4 is produced by astrocytes and released into the brain parenchyma. In ApoE4 carriers, astrocytes and glial cells become reactive, and the MMP9 pathway is activated, leading to increased neuroinflammation. Additionally, the ApoE4 isoform activates the neuronal c/EBPβ pathway, contributing to neuroinflammation, and promotes amyloid and tau pathology independently. It also affects Aβ aggregation by decreasing Aβ lipidation, resulting in increased plaque formation and accumulation. Finally, ApoE4 causes BBB breakdown by disrupting multiple pathways that regulate basement membrane formation, pericytes, and endothelial cells.

In AD, ApoE4 is associated with thin basement membranes and breakdown of the BBB [56]. Furthermore, the ApoE4 allele is associated with diminished capacity to clear interstitial fluid and soluble proteins from the brain, as well as impaired pericyte function that supports adjacent endothelial cells [56,57]. Mice expressing human ApoE4 had a disrupted BBB that was associated with reduced tight junction protein expression, increased dextran permeability indicative of a leaky BBB, increased matrix metallopeptidase 9 (MMP9), and reduced astrocytic support and coverage to blood vessels, compared to mice carrying the ApoE2 or ApoE3 isoforms [58]. Selectively knocking out astrocytic ApoE4 restored most of the BBB functionality; however, mice with complete knock out of ApoE, regardless of isoform, from astrocytes showed some MMP9 activation and BBB dysfunction, suggesting that complete loss of ApoE has negative consequences on the BBB [58]. Moreover, carrying the ApoE4 isoform impairs the BBB ability to repair itself post-traumatic brain injury [59]. In WT mice, the BBB becomes highly permeable after traumatic brain injury [59]. Mice with the ApoE4 genotype showed delayed BBB repair; they exhibited sustained elevated MMP-9 activity, reduced tight junction proteins, and delayed pericyte recovery after injury, highlighting that ApoE4 not only disrupts the BBB but also impairs vascular repair [59]. Further evidence from human iPSC-derived BBB models showed that ApoE4 disrupts the BBB through pericytes [60]. In the ApoE4 iPSC-derived BBB (iBBB), comprising endothelial cells, pericyte-like mural cells, and astrocytes, ApoE4 elevates Aβ accumulation within the iBBB. However, this enhancement was observed exclusively in ApoE4/E4 mural cells, indicating that homozygous ApoE4 genotype mural cells are essential for the ApoE4-associated increase in the Aβ phenotype [60]. Furthermore, RNA sequencing of pericytes indicated a disruption of the calcineurin–nuclear factor of activated T cells (calcineurin-NFAT) signaling pathway, observed both in vitro and selectively within pericytes in human post-mortem brain tissues [60]. Furthermore, the inhibition of the calcineurin-NFAT signaling pathway in ApoE4 iBBB via cyclosporine A (CsA) resulted in a reduction in ApoE expression and Aβ aggregation. This underscores the significance of BBB pericytes in ApoE4-mediated amyloid pathology and identifies calcineurin–NFAT signaling as a prospective therapeutic target [60]. Finally, carrying the ApoE4 genotype enhances the accumulation of the extracellular matrix protein Fibronectin (FN1) at the BBB, which interferes with astrocyte-endothelial interactions and modifies several signaling pathways associated with growth factors that are essential for maintaining BBB integrity, specifically vascular endothelial growth factor (VEGF), heparin-binding epidermal growth factor (HBEGF), and insulin-like growth factor 1 (IGF1) [61]. Furthermore, the accumulation of FN1 associated with ApoE4 is linked to AD pathology, suggesting that FN1 deposits could serve as a potential target to enhance BBB function in ApoE4 carriers with AD [61]. Together, these findings demonstrate that ApoE4 plays a complex role in the progression of AD pathology and in the breakdown of the BBB. Figure 1 summarizes the primary changes caused by the ApoE4 in the AD brain.

## 4. Roles of ApoE in Lipid Metabolism and Lipoprotein Composition

ApoE transports lipids between cells and regulates plasma and tissue lipid levels. Population studies show that ApoE isoforms significantly influence plasma lipid variation. ApoE3 and ApoE2 preferentially bind to phospholipid-rich high-density lipoproteins (HDL), while ApoE4 prefers triglyceride-rich very low-density lipoproteins (VLDL). ApoE3 has minimal effect on lipid levels. In contrast, ApoE2 and ApoE4 exert distinct, opposite effects: ApoE2 is linked to higher ApoE and triglyceride levels but lower cholesterol, whereas ApoE4 is associated with lower ApoE but higher cholesterol. These effects stem from functional differences between isoforms. ApoE variants may explain up to 10% of cholesterol variability in the population [62]. Cell-surface heparan sulfate proteoglycans (HSPGs) facilitate the binding and uptake of ApoE-containing lipoproteins, either by transferring them to receptors such as LRP1 or through direct HSPG-mediated internalization [62].

Lipids constitute at least half of the mass of the CNS and comprise up to 80% of the myelin sheath, the specialized insulating membrane surrounding axons [63]. The altered lipid composition of the brain represents a physicochemical property that changes with normal aging. Moreover, significant modifications in lipid composition are observed in neurodegenerative diseases, including in AD brains [64]. Recent lipidomic analyses of cerebral vessels isolated from the middle temporal cortex of postmortem human AD brains have revealed genotype-dependent lipid alterations [65]. The ApoE4 allele was significantly associated with elevated levels of phosphatidylethanolamine (PE) and decreased levels of sphingomyelin (SM) in the cerebrovasculature. These lipid alterations were correlated with Aβ species and sphingolipid composition. Aβ_40_ levels exhibited a negative correlation with total SM, particularly with SM (C16:0), SM (C24:1), SM (C24:2), SM (C24:0), SM (C22:1), and SM (C16:1). Conversely, Aβ_42_ levels demonstrated a positive correlation with total ceramide and phosphatidylcholine (PC) levels, with ceramide (C24:1) showing consistent associations across various vessel types. Most PE species were positively associated with Aβ_40_, whereas PC subspecies were correlated with Aβ_40_ in large vessels and Aβ_42_ in small vessels. Additionally, Aβ_42_ showed a positive association with specific phosphatidylserine (PS) and phosphatidylinositol (PI) subspecies in small vessels. Furthermore, the ApoE4 dosage inversely correlated with total SM, corroborating prior lipidomic studies showing reduced SM levels in ApoE4 carriers compared to non-carriers in AD [66,67]. Inoue et al. conducted a lipidomic profiling study of postmortem AD brains and observed a similar increase in PE and lower SM and PS levels in ApoE4 cerebral vasculature [65]. Moreover, the ApoE4-driven lipid alterations, mainly reduced SM and increased ceramide, were associated with increased Aβ_40_ and Aβ_42_ in the vasculature [65]. These associations indicate that disruption of vascular lipids contributes to ApoE4-related enhancement of amyloid pathology [65]. Multiple studies have identified altered lipid levels associated with ApoE4 expression in AD brain tissues. Yet, despite increasing evidence from lipidomic studies, the specific functions of individual lipids, their compositions, or their mechanisms in AD development through ApoE interactions remain unclear. Future research to understand the biological roles of lipids in ApoE4-mediated AD is essential. Table 1 summarizes the major alterations in lipids observed within the human AD brain expressing ApoE4 [65,66,67,68,69].

The cholesterol metabolism-associated transporter, ATP-binding cassette transporter A1 (ABCA1), regulates CNS ApoE levels and lipidation. ABCA1 deficiency increases Aβ deposition in mice expressing ApoE4, but not ApoE3, suggesting a stronger link between abnormal cholesterol metabolism and AD in the context of ApoE4 [70]. The lipidation profiles of distinct ApoE isoforms play a pivotal role in modulating Aβ clearance dynamics. ABCA1 deficiency increases Aβ deposition in ApoE4 mice as it impairs the crucial cholesterol efflux needed for ApoE lipidation [70]. Conversely, recent findings demonstrate that ApoE isoforms differentially regulate cellular cholesterol homeostasis through receptor-mediated uptake of cholesteryl esters [71]. ApoE3 and ApoE4, which promote higher cholesteryl esters internalization, upregulate ABCA1 while downregulating cholesterol synthesis genes, reflecting compensatory responses to lipid overload. Lipidomic analyses revealed isoform-dependent accumulation of cholesteryl esters and polyunsaturated lipid species (ApoE4 ≥ ApoE3 > ApoE2), leading to lysosomal lipofuscin deposition and neuronal vulnerability, thereby providing mechanistic insight into how ApoE4-linked lipid dysregulation contributes to AD pathology. Consistent with altered lipid homeostasis in AD, CE (20:4), one of the most abundant cholesteryl ester species, was elevated in the CSF of AD patients compared with age-matched controls [71].

Global transcriptomic analyses reveal human-specific ApoE4-driven lipid metabolic dysregulation in astrocytes and microglia, enhancing cholesterol synthesis, matrisome signaling (the network of extracellular matrix proteins and associated regulators), and glial activation, which may contribute to increased AD risk [72]. ApoE isoforms differentially modulate lipid burden in human iPSC-derived microglia, leading to distinct inflammatory cytokine profiles and transcriptional changes linked to cholesterol homeostasis. Lipid overload, particularly via ApoE4, amplifies cytokine secretion in iPSC-derived microglia under lipopolysaccharide (LPS) stimulation, despite similar cholesterol uptake compared to ApoE3. LDL receptor (LDLR) blockade reverses ApoE4-driven lipid uptake but does not fully restore inflammatory signatures, suggesting lipid-independent effects [71]. ApoE interacts with receptors ranging from LDLR to low-density lipoprotein receptor-related protein 1 (LRP1) and triggering receptor expressed on myeloid cells 2 (TREM2) to regulate cholesterol uptake and clearance in microglia, thereby maintaining intracellular lipid homeostasis [73]. In AD brains, particularly in individuals with the ApoE4/E4 genotype, microglia accumulate lipid droplets near Aβ plaques, with their abundance correlating positively with plaque density and tau pathology [74]. ApoE4-driven lipid droplet (LD) accumulation in microglia impairs neuronal sensing, disrupts cholesterol homeostasis, and promotes tau pathology, whereas ApoE2 preserves microglial function, limits inflammation, and enhances Aβ clearance [75]. In mice expressing human ApoE3 or ApoE4, LPS treatment causes hepatic LD accumulation in ApoE4 mice by disrupting cholesterol and phospholipid trafficking, impairing lysosomal and autophagic degradation [76]. Additionally, ApoE4 shifts the LD proteome toward vesicle transport and pro-inflammatory proteins linked to AD, decreasing microglial sensing and clearance of Aβ and worsening tau pathology [76]. Furthermore, astrocytes expressing human ApoE4 showed increased LD accumulation and larger droplet size [77]. These lipid droplets contained higher levels of AD-linked proteins, along with increased inflammatory cytokines, impaired turnover, and increased peroxidation sensitivity, indicating dysregulated immune responses that could contribute to AD pathology [77]. These observations highlight a potential link between ApoE4-driven lipid accumulation and neurodegenerative pathology.

Biological membranes are highly diverse in structure and contain various microdomains enriched in glycosphingolipids [78]. Glycosphingolipids are unique amphipathic molecules that contain a hydrophilic carbohydrate portion and a hydrophobic lipid component. Glycolipid deficiencies in the CNS are significant, as they lead to severe clinical phenotypes [79]. Sulfatide, a class of glycosphingolipids, has been shown to undergo a marked reduction in the brain during the early stages of AD [80]. This reduction occurs via an ApoE-dependent and isoform-specific mechanism. Further corroborating this observation, a decline in sulfatide content and an expansion in ventricular area were additionally noted in ApoE4 knock-in mice. This discovery indicates a mechanistic link among the ApoE genotype, sulfatide metabolism, and the ensuing pathological alterations, potentially implicating the pathogenesis of AD [80]. Furthermore, ApoE3 and ApoE4 have been shown to regulate the transport of monosialotetrahexosylganglioside (GM1) [81]. Zhang et al. showed that both ApoE3 and ApoE4 have a higher binding affinity for GM1 than for cholesterol in vitro [81]. Moreover, both ApoE isoforms are involved in GM1 transport in a cell-type-specific manner, with transport efficiency regulated by ApoE receptor expression. Furthermore, the same investigators found that GM1 clustering in microdomains (also known as lipid rafts) enhances ApoE-GM1 interaction and, consequently, GM1 cellular uptake [81]. These results could explain ApoE’s role in Aβ aggregation by regulating GM1 [81]. The role of ApoE in Aβ aggregation is hypothesized to start early in the pathology, where the astrocyte ApoE4 effect is more evident in the seeding stage of Aβ, where the Aβ half-life is increased compared to astrocytes carrying the ApoE3 [82]. Moreover, ApoE4 plays a minimal role in Aβ accumulation after seeding; therefore, it is proposed that ApoE-targeted therapy should be preventative rather than treatment [83]. In conclusion, those results suggest that ApoE4-related alterations occur early in the pathology. This is supported by ApoE4’s early interaction with intracellular Aβ in neurons [83]. This interaction starts by internalizing ApoE into neuronal cells, in this case, N2a cells, along with Aβ. Once internalized, ApoE prolongs Aβ residence time within neurons, promoting neuronal Aβ accumulation [84]. Another proposed ApoE4-dependent mechanism is that astrocytes’ ApoE4 increases neuronal APP expression in the microdomains [84]. Increased APP localization in microdomains will lead to higher APP cleavage and, consequently, increased Aβ production. This is accomplished by ApoE4 increasing cholesterol transport, thereby expanding the microdomains, while also recruiting the APP, resulting in higher β- and γ-secretase activity and increased Aβ generation [84,85].

Human ApoE is extensively O-glycosylated on serine and threonine residues in a tissue- and cell-specific manner [86]. CNS ApoE exhibits more extensive glycosylation than plasma ApoE. It has been reported that 66.5% of ApoE in CSF is glycosylated, compared with 12.5% in plasma among older individuals, including those with cognitive impairment [87]. Interestingly, the highest percentage of glycosylated forms is associated with ApoE2, followed by ApoE3 and ApoE4, in non-demented older individuals [88]. The degree of glycosylation may alter the interactions between lipids and Aβ, thereby contributing to AD pathogenesis. The most recent larger cohort study further confirmed that ApoE glycosylation profiles are isoform-specific, with a lower extent of ApoE4 glycosylation in CSF but not in plasma [89]. Moon et al. have shown that CSF ApoE glycosylation levels are inversely associated with AD pathology and cognitive function, specifically CSF t-tau, p-tau181, and forgetfulness [90]. These findings may indicate that reduced ApoE glycosylation is associated with more severe pathology, suggesting that ApoE glycosylation may be protective in AD patients [90].

These results provide evidence for the critical role of ApoE isoform-specific regulation of lipid metabolism in both healthy brain function and neurodegenerative pathology. Further elucidation of how ApoE variants differentially regulate lipid homeostasis is essential to gain key insights into AD progression, identify lipid-modifying strategies to halt neurodegenerative processes, and firmly establish lipid metabolism as a critical therapeutic target in AD.

## 5. Role of ApoE4 in Determining Therapeutic Response in AD

ApoE has emerged as a critical factor influencing not only the risk and development of AD but also how patients respond to treatments. Across various therapeutic options, including disease-modifying therapies, symptomatic medications, and metabolic or inflammatory modulators, growing evidence indicates that the ApoE genotype affects both the effectiveness and safety of these treatments [91]. Specifically, patients carrying the ApoE4 often exhibit different pharmacodynamic responses, faster disease progression despite therapy, or a greater risk of adverse reactions [92,93]. On the other hand, some therapies seem slightly more beneficial or better tolerated in ApoE4 carriers compared to non-carriers [93,94]. These genotype-dependent differences have led to stratified analyses in clinical trials, revised risk–benefit assessments, and, in some cases, genotype-specific therapeutic strategies [95,96]. This section reviews how the ApoE genotype has influenced outcomes across major treatment categories, starting with amyloid-targeting monoclonal antibodies, then moving on to other classes where genotype-stratified responses have been observed [97].

### 5.1. Monoclonal Antibody Therapies

**Figure 2:**
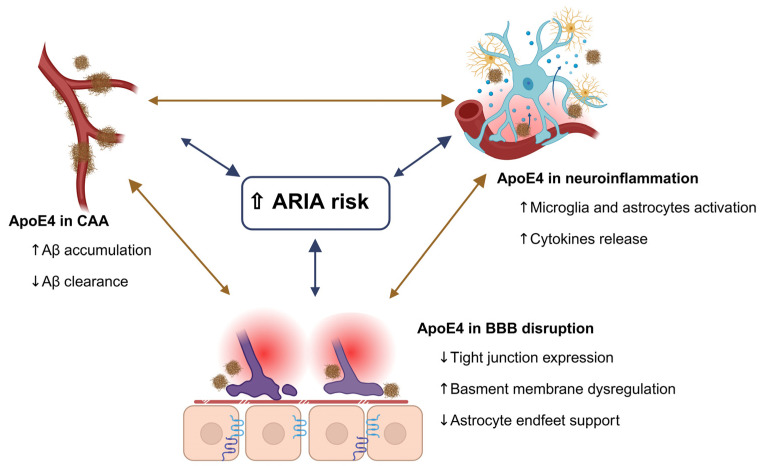
ApoE4 isoform increases the risk of ARIA. The proposed mechanisms explain the increased risk of ARIA in ApoE4 treated with amyloid immunotherapy, including increased neuroinflammation, increased CAA, and increased BBB disruption. The arrows indicate a bidirectional relationship.

Monoclonal antibody therapies targeting Aβ, the new disease-modifying immunotherapies, demonstrate that ApoE genotype influences treatment efficacy. Monoclonal antibodies targeting Aβ (such as aducanumab, lecanemab, and donanemab) have shown the ability to clear Aβ and slow cognitive decline [98,99]. However, ApoE4 carriers show a different safety profile with these agents [100]. ApoE4 carriers experience higher rates of amyloid-related imaging abnormalities (ARIA), edematous or hemorrhagic brain MRI changes associated with anti-Aβ treatment [100,101]. Analyses of clinical trial studies demonstrate that ARIA-E (edema) and ARIA-H (microhemorrhage) events are 3–4 times more frequent in ApoE4 carriers than in non-carriers [100]. ApoE4 increases the risk of ARIA through multiple proposed mechanisms, including effects on BBB integrity, neuroinflammation, and vascular Aβ accumulation. For example, ApoE4 damages the BBB by weakening endothelial tight junctions, promoting basement membrane breakdown via the LRP1-MMP9 signaling pathway, reducing pericyte support, and reducing astrocytic endfeet coverage. Together, these effects weaken BBB integrity and increase the risk of microhemorrhage or edema during anti-Aβ immunotherapy [18,101,102]. Furthermore, ApoE4 promotes an inflammatory state marked by altered microglial and astrocytic activation, elevated pro-inflammatory cytokines, and activation of immune pathways such as TREM2, which can exacerbate vascular injury when amyloid is mobilized by anti-Aβ immunotherapy [101,103]. Additional studies have shown that ApoE4 promotes early Aβ aggregation and reduces its clearance, leading to increased CAA, weakening of blood vessel walls, and impaired perivascular drainage. During antibody-mediated Aβ removal, these abnormalities contribute to the retention of antibody-Aβ complexes within the vasculature, further destabilizing vessels and triggering ARIA [101,104,105]. These proposed mechanisms are depicted in Figure 2.

This tendency of ApoE4 carriers to ARIA has necessitated genotype-tailored dosing protocols and enhanced monitoring. Yet, these immunotherapies remain an essential emerging medication for AD treatment. Accordingly, understanding ApoE-mediated differences and their risk–benefit profile is critical for optimizing therapy on an individual basis. Below, we discuss ApoE-related findings concerning monoclonal antibody treatment. Although some have been discontinued, our focus remains on ApoE4-associated effects regardless of their present clinical application status.

#### 5.1.1. Aducanumab

While the efficacy and clinical manifestations of aducanumab were evaluated in multiple clinical studies, we will review those that assessed the role of the ApoE isoform on treatment effectiveness [106,107,108]. In the EMERGE and ENGAGE Phase 3 clinical trials (n = 3285 patients), aducanumab was tested in early AD, with approximately 65–69% of participants being ApoE4 carriers [109]. While EMERGE exhibited modest cognitive benefit at the 10 mg/kg dose and ENGAGE did not, no significant differences in treatment efficacy were observed between ApoE4 carriers and non-carriers. In contrast, the safety profile outcomes are affected by genotype status. Individuals who received aducanumab (10 mg/kg) experienced adverse reactions, including ARIA-E events, in approximately 35.2% of cases. The incidence was higher in ApoE4 carriers (43.0%) than in non-carriers (20.3%). Moreover, several ARIA-E events occurred in nearly one-third of affected patients. These results highlight that although the ApoE genotype does not alter efficacy, ApoE4 carriers are at significantly higher risk of ARIA adverse reactions, supporting the use of genotyping for risk stratification and monitoring [109]. It is worth noting that Biogen announced the discontinuation of aducanumab in 2024 due to low efficacy [106].

#### 5.1.2. Lecanemab

Phase III Clarity AD clinical trial demonstrated that lecanemab significantly slowed cognitive and functional decline in patients with early AD [110]. The ApoE genotype did not affect overall efficacy; both ApoE4 carriers and non-carriers demonstrated significant improvement compared to the placebo on the primary endpoint and all key secondary measures. Among the treated group, 16% were ApoE4 homozygotes, 53% heterozygotes, and 31% non-carriers. Although numerically greater benefits were observed in non-carriers (~41% slowing) compared to carriers, these differences were not statistically significant, and biomarker responses supported target engagement across all genotypes. Thus, ApoE status did not significantly affect the therapeutic efficacy [110]. However, safety profile outcomes, especially ARIA, were genotype dependent. ARIA (combined ARIA-E and ARIA-H) occurred in 45% of ApoE4 homozygotes on lecanemab versus 19% of heterozygotes and 13% of non-carriers (compared with 22%, 9%, and 4%, respectively, in placebo groups). Severe ARIA-E was highest in homozygotes (5%), while non-carriers did not show any. Likewise, severe ARIA-H was more frequent in homozygotes (13.5%) than in heterozygotes (2.1%) or non-carriers (1.1%). Despite these severe adverse reactions, current therapeutic management recommendations do not differ by genotype. These findings reinforce the utility of ApoE genotyping in risk stratification: while clinical benefit is consistent across groups, ApoE4 homozygotes face substantially higher ARIA risk and require close monitoring during treatment [110].

#### 5.1.3. Donanemab

Donanemab was also evaluated in early symptomatic AD in the Phase II TRAILBLAZER-ALZ trial (n = 257 patients) and the Phase III TRAILBLAZER-ALZ 2 trial (n = 1736 patients) [111]. Approximately 70% of the participants were ApoE4 carriers. Donanemab reduced cognitive and functional decline by ~30% overall; however, post hoc analyses revealed that efficacy varied by ApoE genotype. ApoE4 non-carriers experienced the greatest treatment benefit, while ApoE4 carriers showed little clinical improvement. Thus, ApoE4 status, particularly homozygosity, plays a role in the attenuation of treatment response [111]. On the other hand, the safety profile was also strongly genotype dependent. ARIA-E occurred in ~24% of treated patients, but the rate increased with the presence of ApoE4, with 15% in non-carriers, ~24% in heterozygotes, and 41.7% in homozygotes. Symptomatic and severe ARIA events, including cerebral edema and hemorrhages, were more common in ApoE4 carriers. Notably, three ARIA-related deaths were reported in the Phase III trial; two of them were in ApoE4 carriers with edema, and one with hemorrhage [111,112].

#### 5.1.4. Other Monoclonal Antibodies

While some other monoclonal antibodies targeting Aβ, such as solanezumab, gantenerumab, bapineuzumab, and crenezumab, have been studied in large-scale clinical trials in AD, none of these antibodies demonstrated significant clinical effectiveness, nor did they show any ApoE genotype-dependent treatment benefits.

Solanezumab was assessed in three Phase III EXPEDITION trials, including over 3300 patients, with 66–69% being ApoE4 carriers [113]. Across all trials, solanezumab failed to significantly slow cognitive or functional decline in either ApoE4 carriers or non-carriers. Subgroup analyses revealed no significant interaction between ApoE genotype and treatment efficacy [113,114]. Moreover, gantenerumab, a high-affinity IgG1 antibody targeting aggregated Aβ, was studied in the Phase III SCarlet RoAD and Marguerite RoAD trials [115,116]. Both were terminated early due to futility, and no cognitive benefit was observed in either ApoE4 carriers or non-carriers. Although the trials were underpowered for formal subgroup analyses of efficacy, no differential effect was observed by genotype. However, ARIA-E occurred in 10–15% of treated patients in earlier studies, with a higher incidence in ApoE4 carriers, consistent with broader class effects [116,117].

Crenezumab, an IgG4 isotype antibody thought to engage microglia, was evaluated in two Phase II studies and two halted Phase III trials (CREAD 1 and 2). All failed to meet efficacy endpoints [117]. Although a mild effect was observed in an exploratory subgroup of ApoE4 non-carriers with mild AD in Phase II, it was not confirmed in Phase III. Across the overall population, no genotype-stratified difference in efficacy was reported. Importantly, crenezumab’s safety profile was favorable: ARIA-E incidence was low (<3%) and comparable between ApoE4 carriers and non-carriers [117].

Bapineuzumab was tested in two Phase III trials stratified by ApoE4 status, one in carriers and one in non-carriers, due to prior ARIA risk-averse reactions [118,119]. Both trials failed to demonstrate clinical efficacy, with no significant cognitive or functional benefit observed in either group despite evidence of Aβ and tau biomarker engagement, especially in ApoE4 carriers [118,119]. ARIA-E incidence was notably higher in ApoE4 carriers (9–12%), even at low doses, and further elevated in homozygotes. Non-carriers showed dose-dependent ARIA-E (up to 10% at 1.0 mg/kg) [118,119]. These findings confirmed higher ARIA risk among ApoE4 carriers, but no genotype subgroup experienced a clinical benefit.

### 5.2. Donepezil

ApoE genotype status has been studied in relation to standard AD treatments, specifically acetylcholinesterase inhibitors (AChEIs) such as galantamine, donepezil, and rivastigmine. While these agents are prescribed to manage cognitive symptoms in AD, the extent to which ApoE4 status influences their efficacy remains an area of ongoing investigation. Meta-analyses of several clinical trials have found no statistically significant effects of ApoE genotype on treatment efficacy outcomes [120]. Moreover, a 2022 systematic review examining predictors of AChEI response among 32 studies concluded that “most studies did not find an effect of ApoE status on cognitive response” [120]. Likewise, a 2018 meta-analysis that included 38 studies (RCTs, cohort, and case–control designs) found no significant association between ApoE4 carrier and treatment efficacy. Specifically, among the 38 studies, 5 favored ApoE4 carriers, four favored non-carriers, and 29 showed no difference. In the pooled analysis of 30 studies, the standardized mean difference (SMD) in cognitive response between carriers and non-carriers was 0.022, indicating no significant genotype effect [121]. Supporting this, a pooled analysis of three randomized controlled clinical trials with a total of 335 patients treated with donepezil for 12 weeks found cognitive improvements in both ApoE4 carriers and non-carriers, with ADAS-Cog score changes of −2.95 and −4.09, respectively (*p* = 0.23) [122]. Moreover, the 2018 review by Cheng et al. also noted a possible trend toward greater cognitive response in ApoE4 carriers with longer treatment durations (>9 months), though this did not show statistical significance [121]. While most available studies suggest that ApoE4 does not significantly affect AChEI treatment efficacy at the population level, specific subgroups (MCI patients or long-term responders) may exhibit subtle genotype-associated differences.

### 5.3. NSAIDS

Non-steroidal anti-inflammatory drugs (NSAIDs) have been studied as potential preventive agents in AD, based on their ability to inhibit cyclooxygenase (COX) enzymes and subsequently reduce neuroinflammation, a key pathological feature in AD [123]. Observational studies initially suggested that chronic NSAID use might lower the risk of developing AD, with some data indicating a stronger protective effect in individuals carrying the ApoE4 allele, who may be more vulnerable to inflammation-driven neurodegeneration [124,125]. The majority of these prospective studies demonstrated greater benefits in those with ApoE4 carriers [124,125]. In a randomized controlled trial, the ApoE4 carriers treated with ibuprofen were the only group without cognitive decline [126]. Similar positive effects in ApoE4 carriers have also been observed when NSAIDs have been taken in combination with vitamin E [127]. In addition, NSAID use has been shown to influence microglial activation across all ApoE genotypes; however, the trend toward lower glial cell counts with regular NSAID use was more pronounced in patients carrying the ApoE4 allele. This hypothesis led to large-scale prevention efforts, notably the Alzheimer’s Disease Anti-Inflammatory Prevention Trial (ADAPT), which enrolled approximately 2500 cognitively normal older adults, about 67% of whom had a family history of dementia, to assess whether naproxen or celecoxib could reduce the risk of AD [128]. In the ADAPT, neither naproxen nor celecoxib prevented dementia or slowed cognitive decline, and due to safety concerns, the trial was terminated early [129]. Importantly, ApoE genotype did not influence treatment outcomes; there was no statistically significant interaction, indicating that ApoE4 carriers experienced either greater or lesser benefit from NSAID use [129]. Similarly, the Alzheimer’s Disease Cooperative Study (ADCS) trial examining rofecoxib and low-dose naproxen found no slowing of progression across any ApoE subgroup [130]. Although some observational cohort studies continued to report reduced AD risk in NSAID users with the ApoE4 allele [125], concerns regarding confounding variables and selection bias limit the validity of these associations. Accordingly, these null findings from randomized trials and the absence of genotype-specific efficacy strongly suggest that NSAIDs are not effective for AD prevention or treatment in ApoE4 carriers. The current consensus is that any potential protective effects would likely require administration decades before symptom onset, if they exist at all.

### 5.4. Statins

Statins (HMG-CoA reductase inhibitors), widely prescribed for hyperlipidemia, have been explored for their potential protective and therapeutic roles in AD, with particular focus on differential efficacy by ApoE genotype [131]. Given ApoE’s essential role in lipid metabolism and its link with elevated mid-life cholesterol levels, ApoE4 carriers were hypothesized to derive greater cognitive benefit from statin therapy. While randomized controlled trials, such as an 18-month atorvastatin trial in AD, have not demonstrated significant overall cognitive improvements, re-analysis of patient-level data from previously negative trials revealed a trend suggesting that simvastatin may slightly slow cognitive decline, specifically in ApoE4 carriers [132,133]. Observational findings further support this genotype-specific benefit: across multiple long-term cohorts, statin users, especially ApoE4 carriers, showed better baseline cognitive performance and slower progression of cognitive symptoms over time [132,134]. A large longitudinal cohort study of 4807 participants demonstrated that statin treatment was associated with a significantly reduced risk of AD, with the association markedly stronger among ApoE4 carriers than non-carriers. Among carriers, statin use was also associated with a slower decline in global cognition and episodic memory, an effect not observed in non-carrier individuals [135]. While a 2018 meta-analysis of 30 studies found no significant overall difference in statin response between ApoE4 carriers and non-carriers, a trend toward greater benefit in carriers over longer durations suggests the possibility of genotype-specific effects. Nevertheless, inconsistencies across studies and the lack of definitive disease-modifying outcomes have precluded the use of the ApoE genotype to guide clinical statin use in AD [133]. Collectively, the accumulating evidence suggests a potential neuroprotective role for statins in AD, with ApoE4 possibly deriving the greatest cognitive benefit, warranting further genotype-stratified trials. In Table 2, we summarize how ApoE isoforms affect the effectiveness of drugs discussed in this section. We also share our recommendations based on the existing literature. However, since most studies linking ApoE isoforms to drug effectiveness are observational, they are insufficient to establish therapeutic guidelines, as no recommendations for ApoE genotyping have yet been established.

## 6. Conclusions

AD is characterized by multiple pathological hallmarks, primarily Aβ aggregation, NFT formation, neuroinflammation, and BBB breakdown. One of the primary non-modifiable risk factors includes the ApoE4 isoform. In this review, we discussed recent findings regarding the role of ApoE in driving AD-related pathologies. First, we examined the influence of peripheral ApoE on AD pathology. It has long been established that the peripheral and central ApoE pools are segregated by the BBB. However, recent studies demonstrate that peripheral ApoE not only induces central alterations but can also traverse the BBB, raising the question of how peripheral ApoE can be targeted to mitigate AD pathology. Subsequently, we review new findings concerning the role of ApoE in Aβ aggregation, tau pathology, neuronal death, and neuroinflammation, with particular emphasis on the impact of the ApoE4 isoform on BBB integrity. Given that ApoE is heavily involved in lipid metabolism, especially cholesterol, we discuss lipid alterations associated with carrying the ApoE4 isoform and their consequences, notably those related to APP processing, neuroinflammation, and disease progression. Finally, we conclude our review by exploring how the ApoE4 isoform may influence or suggest alternative therapeutic approaches for AD, in which the outcomes of both AD-specific treatments, such as monoclonal amyloid antibodies, and non-AD therapies, including statins and NSAIDs, may vary depending on the ApoE isoform. The differences in treatment outcomes across ApoE isoforms highlight the importance of precision medicine, in which a patient’s specific genotype dictates the optimal treatment. This approach will yield better outcomes, with greater effectiveness and fewer adverse effects. Overall, these insights emphasize the importance of integrating ApoE genotype clinical decision-making to prevent and treat AD.

In conclusion, this review overviews pathways explaining the role of ApoE4’s in driving AD pathology. Although we have a vast body of research evaluating ApoE, discoveries are essential to understanding pathological differences in an ApoE-dependent manner and to identifying new therapeutic targets in AD. Importantly, these emerging insights highlight ApoE4 not merely as a genetic risk marker but as an active driver of disease progression. A clearer understanding of these mechanisms is essential for developing more precise, patient-derived ApoE-related therapeutic strategies and for explaining the heterogeneous responses to current and emerging AD treatments. In addition, from the available Omics data, it would be essential to understand the differences in AD-altered pathways between ApoE2, ApoE3, and ApoE4 carriers, not only for therapeutic targeting but also to clarify their roles in the disease and identify those who will respond to the treatment.

## Figures and Tables

**Table 1:** Major lipid alterations in the human AD brain of ApoE4 carriers. Abbreviations: PE: Phosphatidylethanolamine PS: Phosphatidylserine SM: Sphingomyelin LPE: Lysophosphatidylethanolamine PC: Phosphatidylcholine FFA: Free Fatty Acid LPC: Lysophosphatidylcholine PI: Phosphatidylinositol PA: Phosphatidic Acid.

Lipid	Change	Source/Region	Ref.
PE	↑	cerebrovasculature	[65]
PS	↓	cerebrovasculature	[65]
SM	↓	cerebrovasculature	[65]
LPE	↑	brain-derived extracellular vesicles	[69]
PC	↑	brain-derived extracellular vesicles	[69]
PE	↑	brain-derived extracellular vesicles	[69]
FFA 20:3	↑	brain-derived extracellular vesicles	[69]
FFA 18:2	↑	brain-derived extracellular vesicles	[69]
FFA 12:0	↑	brain-derived extracellular vesicles	[69]
LPC	↓	right inferior parietal lobule	[68]
LPE	↓	right inferior parietal lobule	[68]
PC	↓	right inferior parietal lobule	[68]
PE	↓	right inferior parietal lobule	[68]
PI	↓	right inferior parietal lobule	[68]
PS	↓	right inferior parietal lobule	[68]
PA	↓	right inferior parietal lobule	[68]
SM	↓	right inferior parietal lobule	[68]
Sulfatide	↓	right inferior parietal lobule	[68]
Cholesterol	↑	right inferior parietal lobule	[68]
SM	↓	middle frontal gyrus	[67]
Ceramide	↑	middle frontal gyrus	[67]

**Table 2:** Influence of ApoE allele on treatment efficacy and safety in AD, along with our suggestions and potential considerations for genotype-guided therapy.

Drug/Class	Effect of ApoE	Suggestions/Considerations
Aducanumab	ApoE genotype did not affect overall efficacy. ARIA-E was detected in 35.2%, with 43% in ApoE4 carriers and 20.3% of non-carriers.	Genotype-guided risk assessment
Lecanemab	ApoE genotype did not affect overall efficacy. ARIA occurred in 45% of ApoE4, and in 13% of non-carriers. Severe ARIA-E was highest in ApoE4/4 (5%) compared to non-carriers (0%).	Genotype-guided risk assessment
Donanemab	Treatment efficacy varies by ApoE genotype. ApoE4 non-carriers experienced the greatest treatment benefit. ARIA-E occurred in ~24%, with 15% in ApoE4 non-carriers, ~24% in one ApoE4 allele carriers, and 41.7% in ApoE4/4 carriers.	Genotype-guided risk assessment
Donepezil/AChEIs	No significant effects of ApoE genotype on treatment efficacy outcomes.	Standard treatment for all genotypes; no genotype-based treatment or dose adjustment required
NSAIDs	ApoE genotype did not influence treatment outcomes.	Not recommended for AD prevention or treatment based on current evidence; genotype does not alter outcomes
Statins	There is a trend suggesting that simvastatin may slightly slow cognitive decline, specifically in ApoE4 carriers. Observational findings support genotype-specific benefits.	Potential neuroprotective role for statins in AD among ApoE4 carriers.

## Data Availability

No new data were created or analyzed in this study. Data sharing is not applicable to this article.
